# Assessment of Voltage Influence in Carbon Dioxide Fixation Process by a Photo-Bioelectrochemical System under Photoheterotrophy

**DOI:** 10.3390/microorganisms9030474

**Published:** 2021-02-25

**Authors:** Sara Díaz-Rullo Edreira, Silvia Barba, Ioanna A. Vasiliadou, Raúl Molina, Juan Antonio Melero, Juan José Espada, Daniel Puyol, Fernando Martínez

**Affiliations:** 1 Department of Chemical and Environmental Technology, ESCET, Universidad Rey Juan Carlos, Móstoles, 28933 Madrid, Spain; sara.diazrullo@urjc.es (S.D.-R.E.); silvia.barba@urjc.es (S.B.); raul.molina@urjc.es (R.M.); juan.melero@urjc.es (J.A.M.); daniel.puyol@urjc.es (D.P.); 2 Department of Environmental Engineering, Democritus University of Thrace, 67100 Xanthi, Greece; ioavasil@env.duth.gr; 3 Department of Chemical, Energy and Mechanical Technology, ESCET, Universidad Rey Juan Carlos, Móstoles, 28933 Madrid, Spain; juanjose.espada@urjc.es

**Keywords:** bioelectrochemical system (BES), Purple Phototrophic Bacteria (PPB), carbon dioxide fixation

## Abstract

Bioelectrochemical systems are a promising technology capable of reducing CO_2_ emissions, a renewable carbon source, using electroactive microorganisms for this purpose. Purple Phototrophic Bacteria (PPB) use their versatile metabolism to uptake external electrons from an electrode to fix CO_2_. In this work, the effect of the voltage (from −0.2 to −0.8 V vs. Ag/AgCl) on the metabolic CO_2_ fixation of a mixed culture of PPB under photoheterotrophic conditions during the oxidation of a biodegradable carbon source is demonstrated. The minimum voltage to fix CO_2_ was between −0.2 and −0.4 V. The Calvin–Benson–Bassham (CBB) cycle is the main electron sink at these voltages. However, lower voltages caused the decrease in the current intensity, reaching a minimum at −0.8 V (−4.75 mA). There was also a significant relationship between the soluble carbon uptake in terms of chemical oxygen demand and the electron consumption for the experiments performed at −0.6 and −0.8 V. These results indicate that the CBB cycle is not the only electron sink and some photoheterotrophic metabolic pathways are also being affected under electrochemical conditions. This behavior has not been tested before in photoheterotrophic conditions and paves the way for the future development of photobioelectrochemical systems under heterotrophic conditions.

## 1. Introduction

The emission of anthropogenic greenhouse gases, particularly carbon dioxide (CO_2_), is considered one of the main causes of climate change and global warming [1,2,3]. Bioelectrochemical systems (BES), which are capable of fixing CO_2_ by using electroactive microorganisms, are considered an environmentally friendly technology that contributes to mitigating carbon footprint concerns [4]. Some microorganisms are capable of receiving electrons from a solid electrode (electrotrophs) [5,6], allowing the development of an electrochemically capable biofilm [7].

Purple phototrophic bacteria (PPB) are a group of microorganisms with a multitude of metabolic pathways [8,9] that have interesting applications in resource recovery from waste sources [10]. This also includes their capacity to uptake external electrons in BES [11,12,13,14]. Some previous works have evidenced the capacity of PPB to uptake electrons and to incorporate them into their metabolism, demonstrating that the main metabolic pathway is the Calvin–Benson–Bassham (CBB) cycle, using the cathode as an electron donor (biocathode) [11,13]. However, these works deal with pure cultures in photoautotrophic conditions, which is not a realistic option for scaling up the process. A recent report has demonstrated that PPB are able to fix CO_2_ at −0.5 V (vs. Ag/AgCl) under photoheterotrophic conditions [12]. However, the minimum voltage necessary to drive the photobioelectrochemical CO_2_ fixation has not been yet studied, either with pure or mixed cultures. 

This work aims to assess the influence of voltage on the CO_2_ fixation process to establish the minimum voltage to accomplish the electroautotrophic process by PPB under photoheterotrophic conditions. These data contribute to the design of bioelectrochemical devices in terms of the energy requirements of the process. Moreover, these results are needed for technoeconomic evaluation of the process, which can enable further scaleup of the technology.

## 2. Materials and Methods

### 2.1. Preparation of Synthetic Media

L-Malic acid and ammonium chloride were used as carbon and inorganic nitrogen sources at 760 mg chemical oxygen demand (COD)/L and 300 mgN/L, respectively. Ammonium inhibits the hydrogen evolution [12], thus limiting the potential electron acceptors of the process. The oxidation of malic acid in the tricarboxylic acid cycle (TCA) generates CO_2_. This approach allows focusing on the CBB cycle without the need to add CO_2_ externally. Macro and micronutrients were prepared following the Ormerod medium [15]. All chemical compounds used were purchased from Sigma-Aldrich (Merck KGaA, Darmstadt, Germany).

### 2.2. Enrichment of PPB and Biofilm Formation

An inoculum of domestic wastewater was used for the enrichment of a PPB mixed culture according to the method described elsewhere [12]. Active PPB biomass was grown over a graphite bar electrode (Carbosytem S.L.L, Parla, Spain) surface by inoculating 5 mL of the enriched PPB mixed culture with the Ormerod medium in a 0.5 L ISO bottle and submerging the graphite bar inside the bottle. The bottle was illuminated with 850 nm LED lamps as NIR (near-infrared) light source at an average irradiance of 20 W/m^2^ and refreshed weekly for 28 days with the Ormerod media. Enrichment culture was maintained in each cycle to ensure the growth of PPB over graphite bar. A picture of the resulting biofilm is included in Supporting Information (Supplementary information, Figure S1).

### 2.3. Photobioelectrochemical Experiments 

#### 2.3.1. Experimental Setup

The photobioelectrochemical experiments were performed in a glass H-cell as described in [12]. Two chambers with a working volume of 0.5 L were separated by a cation exchange membrane (RALEX MEGA a.s., Straz pod Ralskem, Czechia). The graphite bar (10 × 100 mm) with the grown biofilm was used as cathode and the working electrode, whereas the anode chamber was equipped with a counter electrode of Ti coated with Pt (2.5 µm) of 100 × 20 mm and a 10 × 5 mesh (Inagasa S.A., Barcelona, Spain). A RE-5B Ag/AgCl reference electrode (Prosense, Oosterhout, The Netherlands) was placed into the cathode chamber. The three electrodes were connected to the potentiostat NEV4-V2 (Nanoelectra S.L., Alcalá de Henares, Spain) capable of providing a maximum current of ±100 mA and a compliance voltage of ±10 V. A picture of the setup is provided in Supporting Information (Supplementary information, Figure S2). 

#### 2.3.2. Experimental Design

The experiments of BES with the developed PPB biofilm were designed to assess the effect of different voltages on the metabolism of PPB, especially the CO_2_ fixation. Four consecutive batch photobioelectrochemical experiments were conducted at room temperature by progressively varying the voltage after cycles of 7 days: −0.2, −0.4, −0.6 and −0.8 V respect to the Ag/AgCl reference electrode. Additional control experiments were also performed: (i) a photobiological control (open circuit) with a PPB biofilm formed in a graphite bar to evaluate the contribution of the PPB metabolism in absence of current intensity, and (ii) an electrochemical control (abiotic) at −0.8 V with a graphite bar without the PPB biofilm. 

For all the experiments performed in the BES at different voltages, cathode and anode chambers were filled with the nitrogen source, macro and micronutrient solutions of the Ormerod medium in the cathode and anode chambers. The carbon source was added in the cathode chamber only. Final conductivities were 5.6 and 1.95 mS/cm for cathode and anode chambers, respectively. The headspace of each chamber was flushed with argon. The liquid medium of cathode and anode chambers was refreshed at the beginning of each experiment. During the refreshment, the cathode chamber was cleaned to limit the suspended growth and the formation of a biofilm on the chamber walls. This procedure promoted the growth of the PPB attached over the graphite bar surface. Formation of the active PPB biofilm was evidenced by determining the VIS/NIR (visible and near-infrared) absorption spectra in a routine fashion as described below. The duration of every BES experiment was extended up to 7 days. Liquid and gas samples were taken from the cathode chamber and analyzed periodically to monitor the entire process. Liquid soluble components were measured after filtering with a 0.45 μm nylon filter (Chrodisc filter/syringe, CHMLab, Barcelona, Spain). Likewise, the current intensity of each experiment was monitored throughout the time.

### 2.4. Analytical Methods

The enrichment of PPB mixed culture was evaluated by the accumulation of bacteriochlorophylls using VIS-NIR spectra analyses. The absorption spectra of the mixed culture were recorded between 450 to 950 nm by employing a VIS-NIR spectrophotometer (V-630, Jasco, Madrid, Spain). Total and soluble chemical oxygen demand (COD) and volatile suspended solids (VSS) were determined according to standard methods [16]. Biomass concentration was determined by using a standard curve of optical density at 665 and 850 nm for VSS and bacteriochlorophyll concentration, respectively. The gas composition of the headspace of the cathode chamber was analyzed by gas chromatography (GC) with a thermal conductivity detector (TCD). Analysis of the carbon fixation was performed by measuring the CO_2_ accumulation and the total pressure in the headspace of the cathode chamber according to Vasiliadou et al. [12]. 

## 3. Results and Discussion

**Figure 1:**
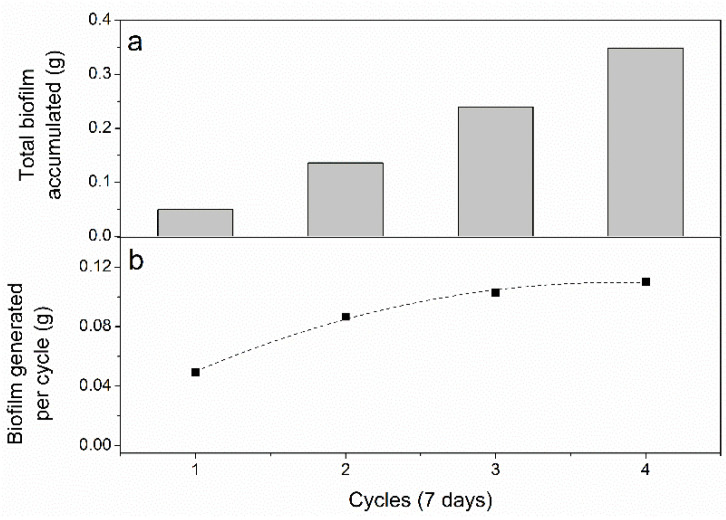
Evidence of active microbial growth over the cathode surface during the bioelectrochemical experiments, as indicated by (**a**) accumulated biomass at the end of each seven-day cycle, and (**b**) biomass generated in each cycle.

The development of an electrochemically capable biofilm plays an important role in the BES to enable the electron transfer between the electrode and the bacteria biofilm [7]. This process is key to maintain a stable CO_2_ fixation under bioelectrochemical conditions [13]. Figure 1 shows the capacity of a mixed culture of PPB to grow under bioelectrochemical conditions in consecutive cycles at increasing voltages. The grams of PPB biomass attached to the cathode after each cycle were calculated as the difference of the estimated production of PPB in terms of volatile suspended solids (assuming a theoretical production of 1 gVSS per 1.73 g of consumed COD per L [8]) and the experimental measurement of volatile suspended solids of the liquid phase. The theoretical basis for this COD balance is included in Supporting Information.

Figure 1 evidenced the increase in the biofilm over the cathode surface as generated per cycle and total grams of biofilm over the graphite bar cathode, which has never been reported before for mixed cultures of PPB. VIS-NIR spectra of this biofilm displayed characteristic peaks at 805 and 865 nm corresponding to bacteriochlorophyll a of PPB [17]. The ratio of absorbance at 805 nm to 600 nm (Abs805/600) is very close to 1.0 (Supplementary information, Figure S3), which has been recently related to a high proportion of PPB in the enrichment [18]. Moreover, these peaks showed a similar magnitude to those observed for the inoculum. It was also seen that the increase in biofilm mass over the electrode achieved an equilibrium after the fourth cycle of seven days, as can be attested from the results of the accumulated biofilm per cycle (Figure 1a). This fact indicates that the growth of the PPB prevails over decay or static mode during the entire working period. Previous experiments determined that PPB decay slowly with a decay rate of 0.09 d-1 [8]. Therefore, it is suggested that the proportion of dead biomass in the cathode did not affect the BES performance. In addition, a direct relationship between eletroactivity of the biofilm and biomass growth was evidenced through cyclic voltametries, where the electroactivity decreased during the course of the experiment as associated with the biomass growth (see Supplementary information, Figure S4). These results reveal that the biofilm was growing under electrochemical conditions, enabling the electron transfer between PPB and cathode. 

**Figure 2:**
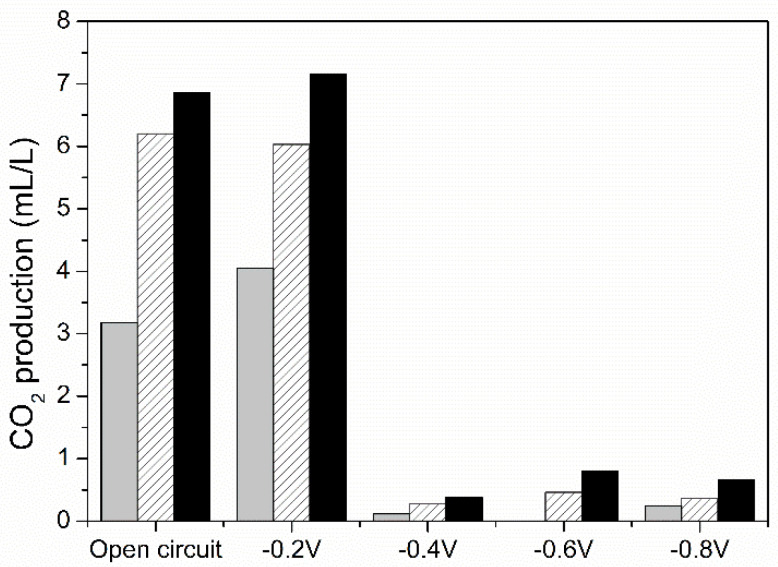
Photoheterotrophic-mediated electrochemical CO_2_ fixation: CO_2_ concentration at one (grey column), two (striped column) and three (black column) days of every experiment at different voltage.

The capacity of PPB to uptake electrons from the electrode has been linked to photosynthetic electron transfer and CO_2_ fixation through the CBB cycle [13]. Figure 2 shows the influence of the voltage on the CO_2_ fixation process. In normal conditions, the malic acid uptake, which is more oxidized than PPB biomass, implies CO_2_ release, as shown for the photobiological control experiment in the absence of the current intensity. In this way, the PPB maintain redox homeostasis [19]. However, under electrochemical conditions, a voltage lower than −0.2 V caused the CO_2_ to be refixed, resulting in no CO_2_ production during the first three days. Therefore, these results confirm that the mixed culture of PPB is capable of bioelectrochemical CO_2_ fixation under photoheterotrophic conditions at a voltage below −0.2 V. 

**Figure 3:**
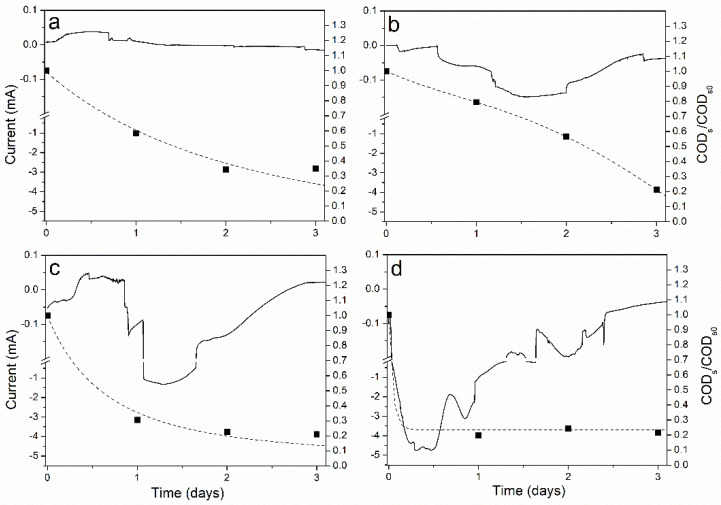
Cronoammperograms (black continuous line) and time course of normalized soluble chemical oxygen demand (COD) (black dash line) at different voltages: −0.2 V (**a**), −0.4 V (**b**), −0.6 V (**c**) and −0.8 V (**d**).

As previously discussed, the CO_2_ fixation process is associated to external electron uptake by the mixed PPB culture. Thus, Figure 3 shows cronoammperograms of the BES experiments performed at different voltages. A significant negative electron current was actively generated by the phototrophic biofilm when decreasing the voltage below −0.2 V. The abiotic control in absence of the mixed PPB culture at −0.8 V (Supplementary information, Figure S5) evidenced neither current decrease nor COD consumption for the electrochemical run. This fact confirms the biological nature of the negative electron current intensity. This bioelectrochemical interaction means that the electron uptake by PPB is only possible at a voltage lower than −0.2 V, as there was no significant current consumption above −0.4 V (Figure 3) and CO_2_ fixation was only possible at values below −0.2 V (Figure 2). These results evidence that the CBB cycle is the initial sink for the excess of electrons, even for a mixed PPB culture. This is in agreement with recent findings in photoautotrophic conditions [13], but in this case, this process occurred under photoheterotrophic conditions.

Additionally, the lowering of the voltage below −0.4 V led to the increment of the electrons’ uptake, increasing the negative current intensity in the cathode. The increment of electron consumption by the PPB biofilm at −0.6 and −0.8 V (Figure 3c,d) is evident in comparison to the experiment carried out at −0.4 V (Figure 3b). Evidence of biological-mediated process is found in the reduction of the conductivity of the anode in biological experiments above −0.2 V, but is found neither in the biological experiment at −0.2 V nor in the abiotic control at −0.8 V, as shown in Supporting Information ( S1). This denoted that cations flow through the membrane only in the presence of the electroactive PPB biofilm. Note also that the COD consumption rate at −0.6 V and −0.8 V is higher than that observed at −0.4 V with no detection of CO_2_ (volumetric COD consumption rates of 147, 401 and 432 mg COD L-1 d-1 for −0.4, −0.6 and −0.8 V, respectively). The decrease in the intensity of the electric current in the cathode once all the CO_2_ has been fixed (which is attained at −0.4 V) suggests the CBB cycle is not the only electron sink of this process. Figure 3 also shows that the increase in current is coincident with the COD depletion, and the current decreases over time when the COD has been consumed. Thereby, some heterotrophic metabolic pathways must be enhanced under BES conditions. Heterotrophic metabolic pathways based on acetoclastic methanogenesis are discarded, as methane was not detected in the headspace of the cathode cell [20]. Another metabolic route that can occur under anaerobic conditions with malic acid as the organic substrate is the malolactic fermentation, where malic acid is transformed into lactic acid [17,21], which is not performed by PPB. However, a previous work shown no accumulation of lactic acid within the media under BES conditions [12]. As the COD was consumed along the experiments (Figure 3), malolactic fermentation can be discarded. Thereby, these bioelectrochemical processes must be performed by PPB since all the organic substrate is being assimilated into bacterial biomass. Metabolic routes related to the accumulation of polyhydroxyalkanoates (PHA) might be plausible. It has been recently reported that *R. palustris* can drive the excess of electrons into PHA under photoautotrophic conditions [22]. A further study is currently ongoing to elucidate these mechanisms in photoheterotrophic conditions.

## 4. Conclusions

This work demonstrates the development of a stable biofilm of mixed cultures of PPB capable of bioelectrochemical CO_2_ fixation under photoheterotrophic conditions. A clear increase in the electroactivity of the biofilm was evidenced with the malic acid uptake, but with nonproduction of CO_2_. The minimum voltage to achieve CO_2_ electro-fixation was below −0.2 V. Voltages lower than −0.4 V allow electron capture through the CBB cycle as the main sink for the excess of electrons, although other metabolic pathways are not discarded. Further studies are necessary to know the fate of the excess of electron consumption by the PPB mixed cultures. These results are significant for the development of novel photobioelectrochemical devices, which should account for the energy requirements of the process to be economically feasible.

## Supplementary Materials

Supplementary materials can be found at https://www.mdpi.com/2076-2607/9/3/474/s1.
