# Supplementary material for: Assessment of Voltage Influence in Carbon Dioxide Fixation Process by a Photo-Bioelectrochemical System under Photoheterotrophy
Source: Microorganisms. 2021 Feb 25;9(3):474. doi: 10.3390/microorganisms9030474 (PMC7996144; doi:10.3390/microorganisms9030474)
Supplement: Supplementary file 1 [file microorganisms-09-00474-s001.pdf]

microorganisms

Supplementary Materials: The following are available online at www.mdpi.com/2076-2607/9/3/474/s1,

Assessment of Voltage Influence in Carbon Dioxide Fixation Process by a Photo Bioelectrochemical System under Photoheterotrophy

Sara Díaz-Rullo Edreira [1], Silvia Barba [1], Ioanna A. Vasiliadou [2], Raúl Molina [1]; Juan Antonio Melero [1], Juan José Espada [3], Daniel Puyol [1], Fernando Martínez [1*].

[1] Department of Chemical and Environmental Technology, ESCET, Universidad Rey Juan Carlos, 28933, Móstoles, Madrid, Spain
[2] Department of Environmental Engineering, Democritus University of Thrace, 67100, Xanthi, Greece
[3] Department of Chemical, Energy and Mechanical Technology, ESCET, Universidad Rey Juan Carlos, 28933, Móstoles, Madrid, Spain
[*] Department of Chemical and Environmental Technology, ESCET, Universidad Rey Juan Carlos, 28933, Móstoles, Madrid, Spain. Phone: 914887182; Email: fernando.castillejo@urjc.es

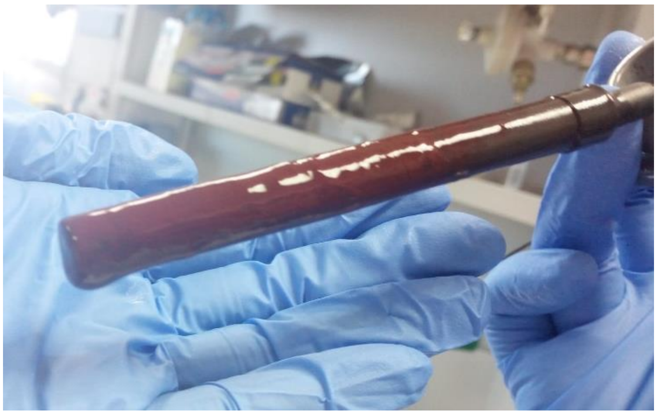

Figure S1. PPB biofilm over the cathode at the end of the biofilm formation.

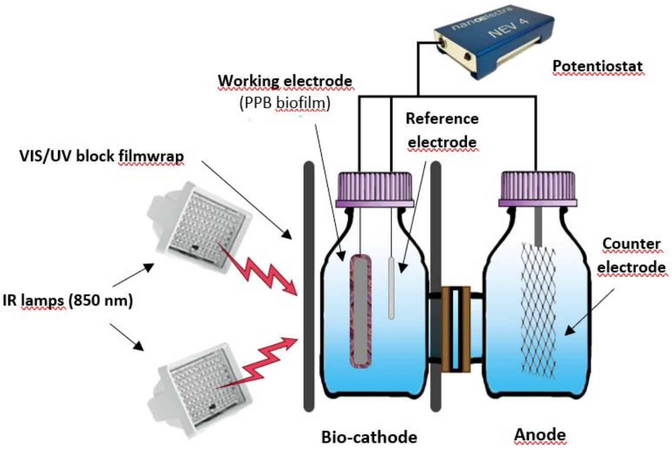

Figure S2. Experimental set-up of the photo-biolectrochemical system.

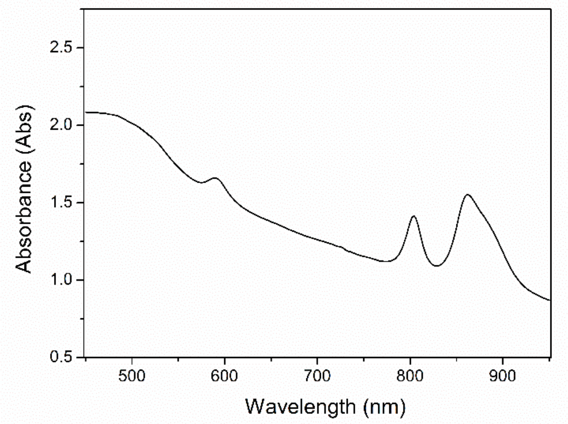

Figure S3. VIS/IR spectra of the biomass attached to the biofilm

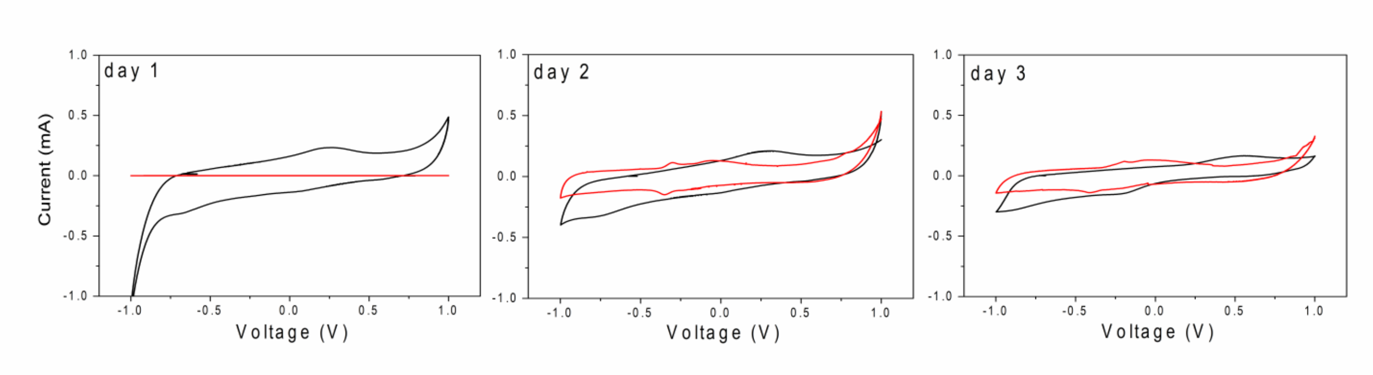

Figure S4. Cyclic voltametries performed during BES experiment (black lines) at -0,8V and the abiotic control (red lines) at -0.8 V and at time = 1 (left), 2 (center) and 3 (right) days.

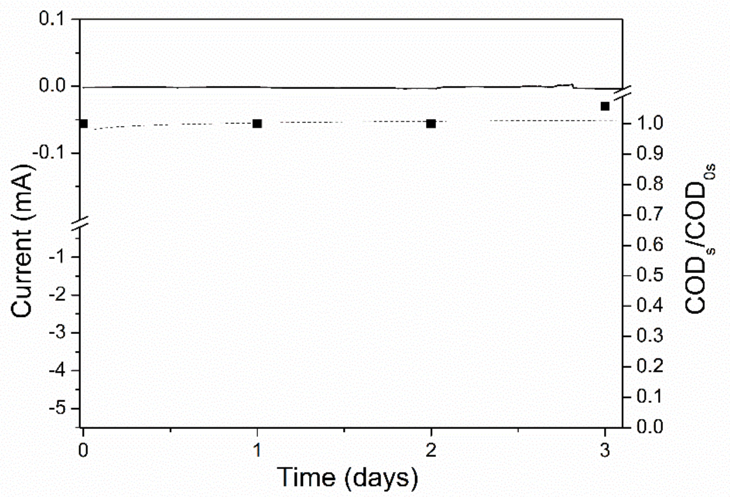

Figure S5. Cronoammperogram (continuous line) and time course of normalized soluble COD (dash line) of electrochemical control (abiotic) at -0.8V.

Table S1. Electrical conductivity (CE) of different experiments (mS/cm)

	-0.2V		-0.4V		-0.6V		-0.8V		Open circuit	Abiotic control -0.8 V	
Day	Cathode	Anode	Cathode	Anode	Cathode	Anode	Cathode	Anode		Cathode	Anode
0	5.60	1.95	5.60	1.95	5.60	1.95	5.60	1.95	5.60	4.42	1.54
1	5.39	2.06	5.59	1.93	5.31	1.92	4.82	1.27	5.32	4.46	2.14
2	5.36	2.23	5.40	1.54	5.32	1.73	4.91	1.42	5.25	6.73	1.77
3	5.24	2.04	5.26	1.46			4.75	1.36		5.30	2.14

S1. Estimation of the biofilm growth by chemical oxygen balances

In order to estimate the accumulation of the biofilm during the bioelectrochemical experiments, we have assumed a homogeneous distribution of the biomass over the biofilm for the estimation of the biofilms growth. We have performed an electron balance (through COD measurements) to accurately predict the biofilm growth over the cathode. In a single batch experiment, the following COD balance can be performed:

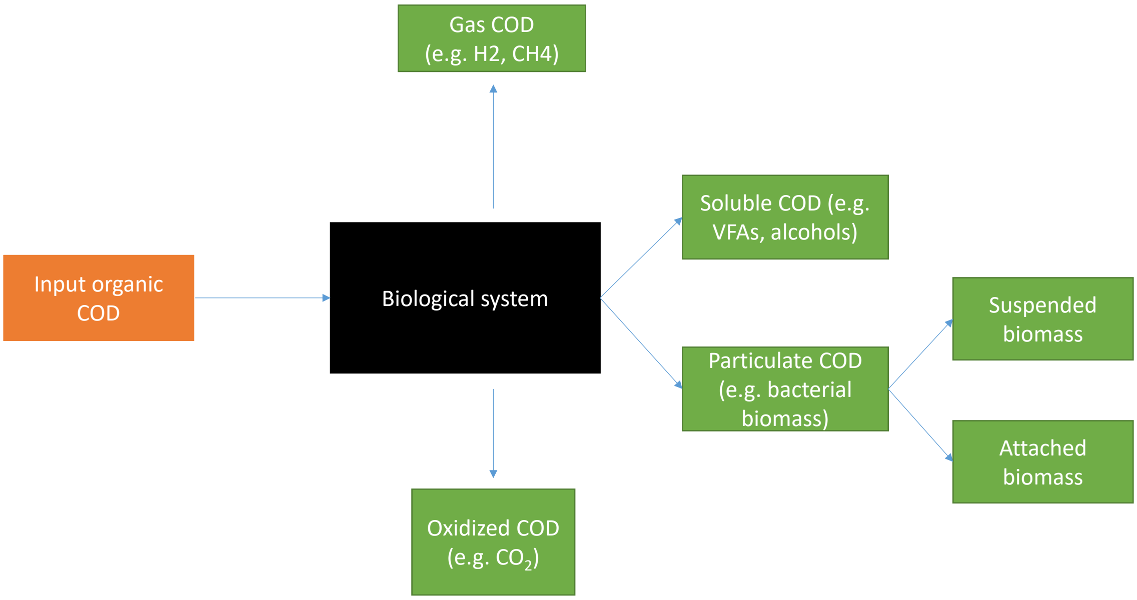

Considering the following:

- Purple phototrophic bacteria assimilate the organic source by using light energy, so no fermentation/oxidation occurs in their metabolism during the uptake of malic acid (e.g. the COD is assimilated into biomass)
- Our system works under anaerobic conditions, with no other oxidized inorganic forms that can accept electrons from the oxidation of biomass (e.g. O_2, NO_2^-, NO_3^-, SO_4^{2-}). Thereby, COD oxidation can be discarded
- There was no measured H2 or CH4 in the headspace, so no COD is lost during methanogenesis or hydrogenogenesis (from dark or photo-fermentation)
- The soluble COD decreased along the experiment, with no production of other volatile fatty acids or alcohols. This discards fermentation processes

Thereby, the conservation of COD obligates to assume that all COD was converted into particulate COD (e.g. biomass). We have measured the suspended particulate COD after brushing the reactor walls at the end of every batch and, in all cases (including the biological control), the COD was not conserved. Therefore, we must assume that the rest of the particulate COD is attached onto the graphite bar.
